# PAC1 Agonist Maxadilan Reduces Atherosclerotic Lesions in Hypercholesterolemic ApoE-Deficient Mice

**DOI:** 10.3390/ijms252413245

**Published:** 2024-12-10

**Authors:** Lilli Mey, Gabriel A. Bonaterra, Joy Hoffmann, Hans Schwarzbach, Anja Schwarz, Lee E. Eiden, Eberhard Weihe, Ralf Kinscherf

**Affiliations:** 1 Department of Medical Cell Biology, Institute for Anatomy and Cell Biology, Medical Faculty, Philipps-University of Marburg, Robert-Koch-Str. 8, 35037 Marburg, Germanyhans.schwarzbach@uni-marburg.de (H.S.); anja.schwarz@staff.uni-marburg.de (A.S.); weihe@staff.uni-marburg.de (E.W.); ralf.kinscherf@staff.uni-marburg.de (R.K.); 2 Section on Molecular Neuroscience, National Institute of Mental Health Intramural Research Program, 49 Convent Drive, Room 5A38, Bethesda, MD 20892, USA; eidenl@mail.nih.gov

**Keywords:** ApoE deficiency, apoptosis, atherosclerosis, atheroprotection, cardiovascular, cyclooxygenase-2, cytokines, inflammation, maxadilan, PACAP

## Abstract

A possible involvement of immune- and vasoregulatory PACAP signaling at the PAC1 receptor in atherogenesis and plaque-associated vascular inflammation has been suggested. Therefore, we tested the PAC1 receptor agonist Maxadilan and the PAC1 selective antagonist M65 on plaque development and lumen stenosis in the ApoE^−/−^ atherosclerosis model for possible effects on atherogenesis. Adult male ApoE^−/−^ mice were fed a cholesterol-enriched diet (CED) or standard chow (SC) treated with Maxadilan, M65 or Sham. Effects of treatment on atherosclerotic plaques, lumen stenosis, apoptosis and pro-inflammatory signatures were analyzed in the brachiocephalic trunk (BT). The percentage of Maxadilan treated mice exhibiting plaques under SC and CED was lower than that of Sham or M65 treatment indicating opposite effects of Maxadilan and M65. Maxadilan application inhibited lumen stenosis in SC and CED mice compared to the Sham mice. In spite of increased cholesterol levels, lumen stenosis of Maxadilan-treated mice was similar under CED and SC. In contrast, M65 under SC or CED did not reveal a significant influence on lumen stenosis. Maxadilan significantly reduced the TNF-α-immunoreactive (TNF-α^+^) area in the plaques under CED, but not under SC. In contrast, the IL-1β^+^ area was reduced after Maxadilan treatment in SC mice but remained unchanged in CED mice compared to Sham mice. Maxadilan reduced caspase-3 immunoreactive (caspase-3^+^) in the tunica media under both, SC and CED without affecting lipid content in plaques. Despite persistent hypercholesterolemia, Maxadilan reduces lumen stenosis, apoptosis and TNF-α driven inflammation. Our data suggest that Maxadilan provides atheroprotection by acting downstream of hypercholesterolemia-induced vascular inflammation. This implicates the potential of PAC1-specific agonist drugs against atherosclerosis even beyond statins and PCSK9 (proprotein convertase subtilisin/kexin type 9) inhibitors.

## 1. Introduction

Atherosclerosis is a chronic inflammatory process of arteries responsible for about 50% of deaths in the Western world [1,2]. Driven by low-density lipoprotein oxidation (oxLDL) and endothelial dysfunction, an inflammatory milieu arises, to which inflammatory leukocytes, mainly monocytes and macrophages (MΦ), are attracted. The presence of pituitary adenylate cyclase-activating polypeptide (PACAP) and its receptors in cardiac tissue and blood vessels was taken as evidence that this neuropeptide may play a role in cardiovascular functions [3]. PACAP, which binds with high affinity to its specific receptor PAC1, protects cultured vascular endothelial cells (EC) and SMC from lipid peroxide-induced damage, which is triggered by elevated lipids [4]. These observations may suggest the involvement of PACAP in attenuating atherogenesis. In fact, using an ApoE^−/−^ mouse model of atherosclerosis we have recently shown that PACAP deficiency aggravates atherosclerosis in mice fed standard chow for 30 weeks [5]. Therefore, PACAP has been suggested to act as an endogenous atheroprotectant [5]. However, constitutive PAC1 deletion in the mouse results in atheroprotection rather than acceleration of atherogenesis [6], a finding at odds with the loss of apparent atheroprotection elicited by PACAP knockout. Since both maneuvers result in lifelong loss of either PACAP or its receptor, and since PACAP does interact with other receptors besides PAC1, we decided to initiate experiments in which gain and loss of PAC1-dependent PACAP function was accomplished pharmacologically, i.e., within the context of reversing atherogenesis, rather than affecting the initial factors predisposing an organism to it.

Interestingly, Maxadilan, a selective agonist for PAC1 isolated from the saliva of sandflies [7], which specifically activates the PAC1 receptor, has been shown to exert anti-apoptotic effects during the downregulation of cleaved caspase-3 [8]. Furthermore, a specific PAC1 antagonist, M65, has been previously engineered based on the Maxadilan peptide backbone [9,10]. Therefore, we tested in the present study our hypothesis that changes in PACAP function in adults, rather than during early development before adulthood, would separate the combined developmental and adult effects of the gain- and loss-of-function of PACAP and its cognate receptor during atherogenesis, providing a more therapeutically relevant assessment of its potential efficacy in the context of progressive cardiovascular disease. We show here that treatment with the PACAP agonist Maxadilan reduced the development of atherosclerosis in ApoE^−/−^ mice, while M65 did not recapitulate the anti-atherogenic effects of PAC1 knockout, indicating that the latter is likely to be a result of developmental dysregulation unrelated to a potential therapeutic effect of PACAP acting at its cognate receptor, in treatment of atherosclerosis.

## 2. Results

The essential new finding of our study is that Maxadilan reduced atherosclerosis in the aortic arch and brachiocephalic trunk of ApoE^−/−^ mice under SC and CED with preserved or further enhanced hypercholesterolemia.

### 2.1. Maxadilan (PAC1 Agonist) i.p., but Not M65 (PAC1 Antagonist) i.p., Reduced Atherosclerotic Plaque Frequency in the Aortic Arch and Its Branches

**Figure 1:**
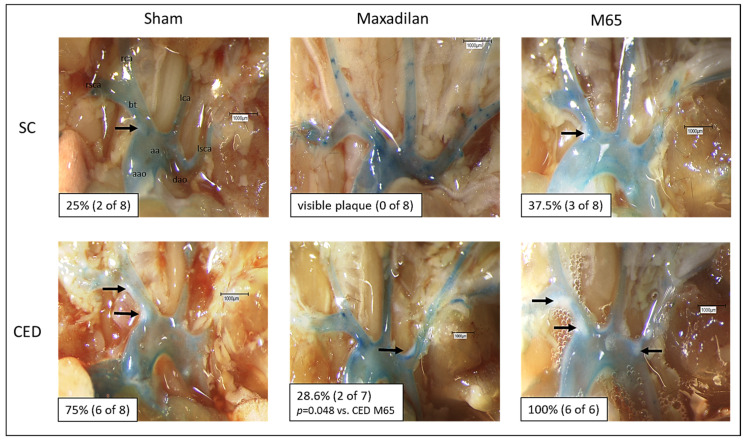
Percentage of mice exhibiting macroscopically visible atherosclerotic plaques in the ascending aorta (aao), descending aorta (dao), aortic arch (aa), and branches of Sham and Maxadilan-treated ApoE^−/−^ mice under standard chow (SC) or cholesterol-enriched diet (CED): Brachiocephalic trunk (BT), right/left carotid artery (rca/lca), right/left subclavian artery (rsca/lsca). Intraluminal contrast staining with methylene blue enhances the visibility of white atherosclerosis plaques, indicated by black arrows. For statistical significance, see *p* values in the individual micrographs. The numbers in parentheses denote the numbers of animals.

En-face dissection revealed that 25% of the ApoE^−/−^ mice under SC developed macroscopically visible atherosclerotic plaques of the aortic arch and its main branches (Figure 1). In contrast, Maxadilan-treated mice revealed no visible plaques (Figure 1). In the CED group, 75% of the Sham mice showed distinct plaques (Figure 1), whereas only 28.6% of the Maxadilan-treated mice developed plaques (Figure 1). In contrast, 37.5% of M65 treated mice under SC revealed atherosclerotic lesions and 100% of M65 treated mice under CED showed atherosclerotic plaques (Figure 1). Thus, M65 is shown to have actions opposite to those of Maxadilan, as it enhances the percentage of mice exhibiting atherosclerotic plaques as compared to the Maxadilan and Sham mice (Figure 1).

### 2.2. Maxadilan (PAC1 Agonist) i.p., but Not M65 (PAC1 Antagonist) i.p., Reduced Lumen Stenosis in BT

**Figure 2:**
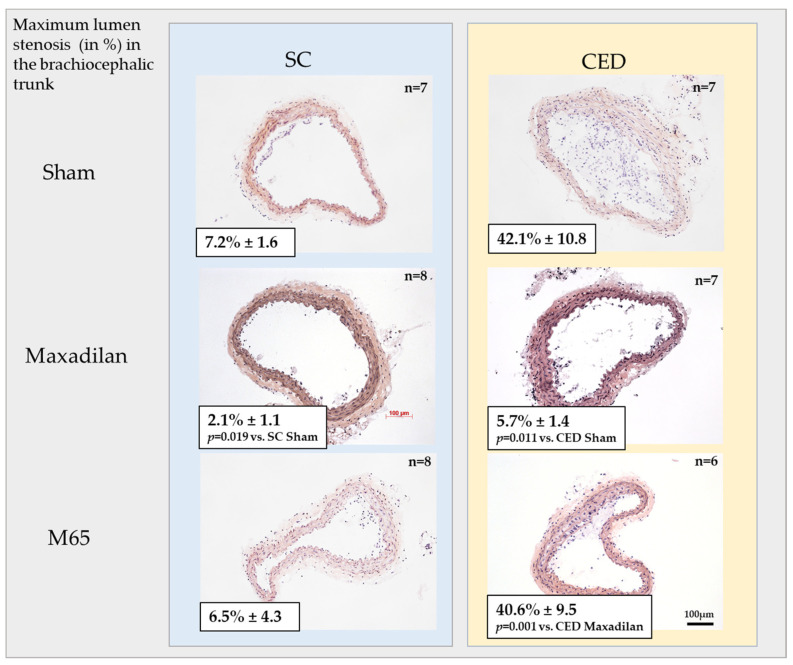
Lumen stenosis and plaque development in the brachiocephalic trunk (BT)of ApoE^−/−^ mice at the age of 20 weeks, fed with standard chow (SC) or cholesterol-enriched diet (CED). Treated mice received PAC1 agonist Maxadilan (20 nmol/kg) or PAC1 antagonist M65 (20 nmol/kg), dissolved in physiological saline solution i.p., Sham groups received physiological saline solution. Cross sections of areas with maximum plaque sizes were prepared and stained with hematoxylin/eosin. Lumen stenosis was measured in %; data are provided as mean ± SEM. For statistical significance, see *p* values in the individual micrographs.

Histomorphometric analysis of cross-sections of BT revealed that Maxadilan treatment of ApoE^−^^/^^−^ mice fed SC significantly (*p* ≤ 0.05) inhibited lumen stenosis in 70% (7.2% vs. 2.1%) of mice compared to the Sham mice (Figure 2). In CED mice, Maxadilan i.p. significantly (*p* = 0.011) reduced lumen stenosis in 86.5% (42.1% vs. 5.7%) animals compared to the Sham group (Figure 2).In spite of significantly increased cholesterol levels, the lumen stenosis of Maxadilan-treated mice fed CEDs and SC was similar (Figure 2; Table 1). In contrast, the application of M65 to mice under SC or CED revealed neither inhibition nor progression of lumen stenosis compared to Sham (Figure 2).

As we have thus shown that Maxadilan, but not M65 treatment, is atheroprotective, we concentrated in the following on the influence of Maxadilan treatment on distinct atherosclerosis-relevant signatures to decipher mechanisms of its atheroprotectant actions.

### 2.3. Maxadilan (PAC1 Agonist) i.p. Decreased Plasma Triglyceride Levels and Increased Total and Free Cholesterol and Body Weight/Tibia Ratio

Under SC, Maxadilan-treated ApoE^−/−^ mice showed 66.9% (*p* ≤ 0.05) lower plasma triglyceride levels than Sham (Table 1). Maxadilan-treated ApoE^−/−^ mice under CED exhibited 37.8% lower triglyceride levels than Sham CED (Table 1). Under SC, Maxadilan-treated ApoE^−/−^ mice showed 33.5% (*p* ≤ 0.05) higher total plasma cholesterol levels than Sham (Table 1). After the differentiation of cholesterol into free cholesterol and cholesterol esters, Maxadilan-treated ApoE^−/−^ mice under SC had 169.0% (*p* ≤ 0.001) higher concentrations of free cholesterol than Sham (Table 1). Remarkably, the atheroprotective effects of Maxadilan treatment occurred despite higher total- cholesterol and free cholesterol-, as well as higher cholesterol ester levels after SC or CED in comparison to Sham. Maxadilan-treated mice revealed significantly 11.2% (*p* ≤ 0.05) or 15.9% (*p* ≤ 0.01) higher body weight/tibia length ratios than corresponding Sham mice under SC or CED (Table 1).

### 2.4. Maxadilan (PAC1 Agonist) i.p. Reduced TNF-α^+^, IL-1β^+^ and Caspase-3^+^, and Increased COX-2^+^ Areas

**Figure 3:**
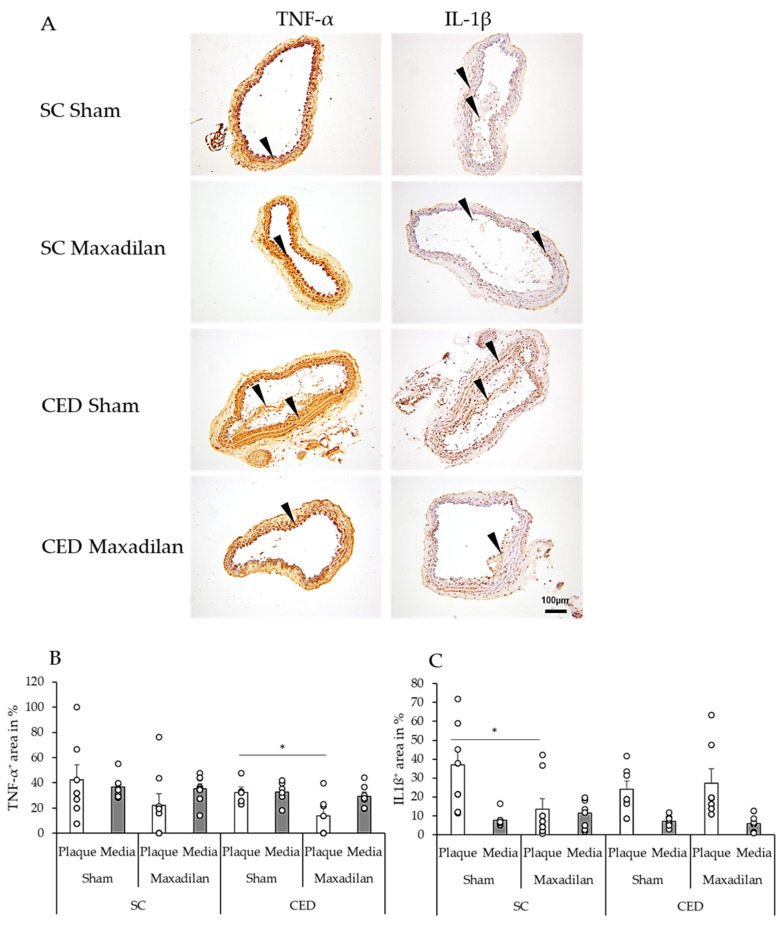
Immunohistochemistry and quantification of the inflammatory markers TNF-α and IL-1β in the plaque and tunica media of BT in ApoE^−/−^ mice under SC and CED, after Maxadilan treatment or Sham (**A**). The percentages of immunohistochemically positive-stained areas per plaque or media were evaluated. Black arrowheads indicate immunoreactive positive cells. Data (**B**) TNF-α and (**C**) IL-1β are provided as mean + SEM (n = 7–9). * *p* ≤ 0.05 vs. Sham under SC or CED. Scale bar: 100 µm.

**Figure 4:**
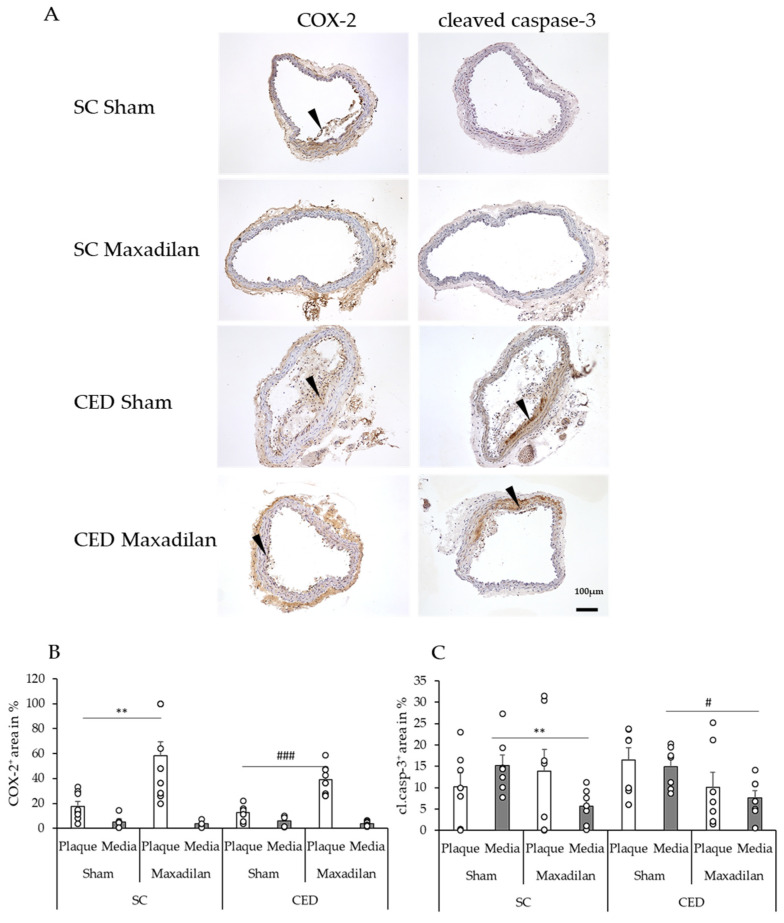
Immunohistochemistry and quantification of immunoreactive areas (in %) in relation to the whole plaque or media of cross sections of BT of ApoE^−/−^ mice under SC or CED after Maxadilan treatment or Sham (**A**). Antibodies were directed against cyclooxygenase-2 (COX-2) and cleaved caspase-3 (cl. casp.3, apoptosis). Black arrowheads indicate immunoreactive positive cells. Data (**B**) COX-2 and (**C**) cleaved caspase-3 are provided as mean + SEM (n = 7–9). ** *p* ≤ 0.01 vs. Sham under SC; ^#^ *p* ≤ 0.05 ^###^ *p* ≤ 0.001 vs. Sham under CED. Scale bar: 100 µm.

Under CED, Maxadilan reduced TNF-α^+^ area in plaques by 18.3% (*p* = 0.04) compared to Sham (Figure 3B). Under SC, Maxadilan-treated mice showed 23.6% (*p* = 0.04) lesser IL-1β^+^ areas in the plaque than Sham. However, under CED, no significant changes in the percentage of IL-1β^+^ areas were observed in the atherosclerotic plaques or SMC of the media. Under SC, Maxadilan treatment increased the COX-2^+^ area in the lesion by 40.4% (*p* ≤ 0.01) and in the CED mice, this was increased by 26.2% (*p* ≤ 0.001) compared to the Sham-treated mice(Figure 4B). Under SC, cleaved caspase-3^+^ cells were found in plaques and media beneath the plaque in BT of Sham ApoE^−/−^ mice (Figure 4C). In the SC mice, the percentage of the cleaved caspase-3^+^ area in the media of Maxadilan-treated mice was decreased by 9.5% (*p* ≤ 0.01), and in CED mice, this was decreased by 7.3% (*p* ≤ 0.05) compared to the Sham mice (Figure 4C).

**Figure 5:**
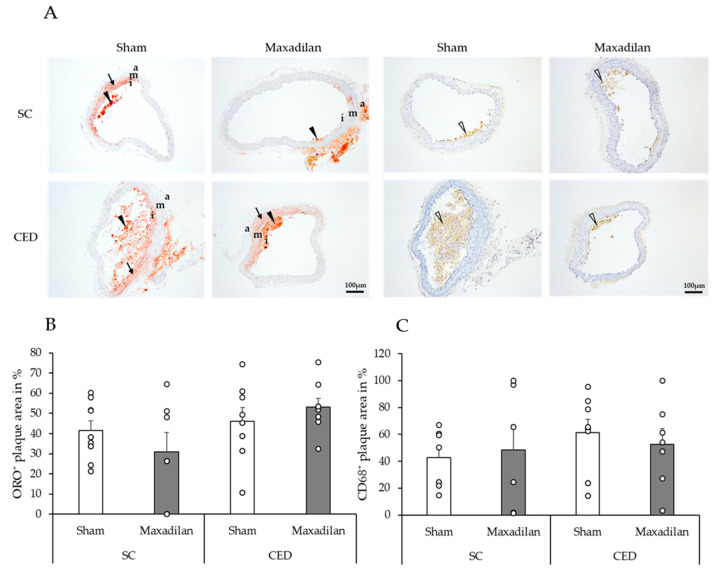
ORO (on the left) and CD68 (on the right) (immuno)histochemistry of atherosclerotic BT cross-sections of ApoE^−/−^ mice under Maxadilan treatment or Sham SC or CED. Oil red positive lipid deposits are shown in red. (**A**,**B**) Lipid-loaden MΦ in the plaque-forming foam cells (black arrowheads) are distinguishable from intimal cells, smooth muscle cells of the tunica media with small lipid droplets (arrows) and cells of perivascular fat tissue showing distinct fat vacuoles. (**C**) CD68^+^ Mo and MΦ (right side, white arrowheads) stained with HRP-DAB in brown. Data are provided as mean + SEM (n = 7–9). Diagrams show % of lipid or Mo/MΦ positive area in relation to the whole plaque area. Intima (i), media (m) and adventitia (a). Scale bar: 100 µm.

Moreover, lipid deposits were detected by Oil Red O (ORO) staining and MΦ were identified by immunohistochemistry using CD68 antibodies (Figure 5A,C). The percentage of ORO plaque area and CD68^+^ MΦ were similar among all groups in the experiment (Figure 5B,C).

## 3. Discussion

Here we have shown that Maxadilan reduces atherogenesis in ApoE^−/−^ mice both, under SC and CED confirming our hypothesis, that PACAP is atheroprotective. In contrast, M65 treatment had no significant effect on lumen stenosis under SC or CED, except that it increased the percentage of mice exhibiting macroscopically visible atherosclerotic plaques compared to Sham and especially to Maxadilan-treated mice. Our present results concord with our previous findings that PACAP deficiency in ApoE^−/−^ aggravates atherosclerosis [5], suggesting that endogenous PACAP is atheroprotective. As Maxadilan is a high-affinity specific PAC1 agonist compared with an extremely low-affinity VPAC2 [11,12], our present study indicates that the atheroprotectant effect of Maxadilan is mediated by the PAC1 receptor. It is worthy of note that a comparison of M65 and Maxadilan effects shown here, resolves a discrepancy between the pro-atherogenic effects of PACAP deletion, and the amelioration of atherogenesis seen in PAC1-deficient mice fed a CED. It has since become clear that knockouts from conception within the PACAP system may have profound developmental effects manifest later in adulthood [13,14]. Thus, gain- and loss-of-function in constitutive knockout mice, at least as regards PACAP signaling, are not inevitably opposite, and in particular, depend on how long the loss (or gain) of function goes on and when it starts. Thus, pharmacological treatment with the PAC1 antagonist M65 did not copy the anti-atherogenic effect of PAC1 deficiency [6]. In contrast, since PACAP binds with high affinity to PAC1, the anti-atherosclerotic effect of the PAC1 agonist Maxadilan perfectly corresponded to the loss of endogenous atheroprotection previously described in PACAP^−/−^/ApoE^−/−^ mice, showing the aggravation of atherosclerosis (as described by Rasbach et al., 2019 [5]), which indicates that PACAP may act as an endogenous atheroprotectant.

In the following, we consider possible mechanisms by which Maxadilan exerts its anti-atherogenic effects.

Pro-inflammatory cytokines such as TNF-α and IL-1β are well-known pro-atherogenic, and inhibiting these cytokines attenuates atherosclerosis [15,16,17]. In fact, anti-TNF-α therapy improves microvascular endothelial dysfunction in rheumatoid arthritis patients with atherosclerosis [18]. Maxadilan has already been shown to act anti-inflammatorily by reducing TNF-α release by LPS-stimulated MΦ [19]. Our findings that Maxadilan also reduces IL-1β are totally novel and underline the anti-inflammatory potential of Maxadilan, which might be of interest for several diseases. Our observation that Maxadilan is atheroprotective in spite of enhanced cholesterol levels in conjunction with the well-known anti-inflammatory effects of PAC1 activation [20] indicates that Maxadilan acts downstream of cholesterol-induced vascular inflammation. The reason why Maxadilan reduces TNF-α^+^ area in the plaque only under CED and IL-1β^+^ areas only under SC remains unclear and needs further investigations. In addition, our findings that Maxadilan reduces hypertriglyceridemia suggest that this effect may also contribute to Maxadilan’s anti-atherogenic action revealed in the present study.

Our results that Maxadilan enhances the percentage of COX-2^+^ areas in atherosclerotic plaques under both, SC and CED deserves particular attention, because it is well-known that COX-2 prostanoid signaling has vasoprotective components [21]. Previously, Maxadilan has been shown to increase the production of PGE2 by MΦ [21] and increased expression of COX-2 reduces atherogenesis in hyperlipidemic mice [22]. Albeit COX-2 is also known as a pro-inflammatory factor [23] the COX-2 inhibitor Celecoxib has been reported to aggravate atherogenesis in ApoE^−/−^ mice [24]. Therefore, we argue that the observed increase of COX-2^+^ areas in the vascular wall seen under Maxadilan treatment could reflect the induction of COX-2-mediated vasoprotection.

Enhanced apoptosis is a characteristic feature of atherosclerosis, albeit with controversial clinical significance [19,25,26,27]. Clarke et al., 2008 [28] conclude that VSMC apoptosis accelerates atherosclerosis, causes calcification and medial deterioration, and promotes stenosis. Most recently, atherosclerosis has been classified as a smooth muscle cell-driven tumor-like disease [29]. The presence of an apoptosis defect in MΦ in mice suggested that apoptosis is a critical self-defense mechanism in inhibiting atherogenesis [30]. Therefore, our findings that Maxadilan reduced cleaved caspase-3^+^ areas in the SMC media layer of the vascular wall suggest that part of the anti-atherogenic effect of Maxadilan is attributable to its anti-apoptotic action. Further studies will be needed to determine whether compounds such as caspase inhibitors will become clinically used to treat patients with vulnerable lesions [31].

Taken together, we conclude that the atheroprotective effect of Maxadilan is mainly downstream of cholesterol-induced inflammation, such as induction of TNF-α and IL-1β and, in addition, may involve activation of vasoprotective signaling, reduction of apoptosis, and hypertriglyceridemia. We suggest that therapeutic approaches with PAC1 agonists under real-world clinical conditions could complement statin and PCSK9 (proprotein convertase subtilisin/kexin type 9) therapy of patients at risk of developing atherosclerosis-related pathologies. Provided that it can be shown in animal models of atherosclerosis that Maxadilan has additive atheroprotective effects when combined with statins or PCSK9 (proprotein convertase subtilisin/kexin type 9) inhibitors, we suggest that therapeutic approaches with PAC1 agonists under real-world clinical conditions could complement clinical therapy with statins and and PCSK9 inhibitors of patients at risk of developing atherosclerosis-related pathologies

## 4. Materials and Methods

### 4.1. Animal Experiments

The parental homozygous ApoE^−/−^ mice were purchased from Jackson Laboratories (B6.129P2-Apoetm1Unc/J, Stock No: 002052|ApoE KO, Jackson Laboratories, Milan, Italy). Mice were bred and genotyped in the animal facilities of the Philipps-University of Marburg. At 10 weeks, male ApoE^−/−^ mice were fed with standard chow (SC; LASQCdiet^®^ Rod16 Rad; LASvendi, Soest, Germany) or a cholesterol-enriched diet [(CED; “Western-type diet”, 21% fat, 0.15% cholesterol and 19.5% casein), catalog number 132010, Altromin GmbH, Lage, Germany]. The long-term intraperitoneal (i.p.) administration of Maxadilan or M65 was performed according to Yu et al., 2008 [32], as were the concentrations utilized. During this period, mice were intraperitoneally (i.p.) injected three times a week with 20 nmol/kg of Maxadilan (4067317, CAS Number: 135374-80-0, Bachem AG, Bubendorf, Switzerland) or M65 (4067721, CAS Number: 1872440-65-79 Bachem AG) diluted in a physiological saline solution (B. Braun, Melsungen, Germany). The animals were distributed into 4 groups: Sham SC (n = 8), Sham CED (n = 8), Maxadilan SC (n = 8), Maxadilan CED (n = 7), M65 SC (n = 8), and M65 CED (n = 6). Sham-treated animals were injected with physiological saline solution alone (B. Braun, Melsungen, Germany). The solutions were freshly generated, kept cool, and administered under sterile conditions. All experiments were approved by the federal state veterinary authority, Regional Commission Gießen (V 54–19 c20 15 h 01 MR 20126; Nr. G 80/2019), and conducted according to the animal experiment care guidelines of GV-SOLAS (Committee for Animal Welfare Laboratory animal husbandry, August 2014, URL, accessed on 11 November 2022, (https://www.gv-solas.de/wp-content/uploads/2021/08/hal_201408Tiergerechte-Haltung-Maus.pdf). General health conditions, behavior, and body weight were monitored during all procedures, and no signs of impairment, suffering, weight loss, or abnormal weight gain were observed. After 10 weeks of treatment, animals were narcotized to Guedel stage III-IV, the thoracic cavity was opened and mice were euthanized by exsanguination from the right atrium. Blood was collected in heparinized microtubes 1.3 mL LH, at 25 I.U. heparin/mL blood (Sarstedt, Nümbrecht, Germany). After the right atrial incision, the vascular system was perfused with phosphate-buffered saline (PBS) solution with 0.01% heparin (B. Braun SE, Melsungen, Germany) through punctation of the left ventricle. The vascular system was injected with 200 μL PBS containing methylene blue (0.25%; Riedel-de Haën, Seelze-Hannover, Germany). The perfused hearts and BTs were removed, frozen in liquid nitrogen-cooled isopentane, and stored at −80 °C. 

### 4.2. Determination of Cholesterol and Triglyceride Levels

Blood samples in heparinized tubes were centrifuged (10 min at 650× *g*), and heparinized plasma was removed and stored at −80 °C. Using a cholesterol/cholesteryl ester quantitation kit (ab65359, Abcam, Cambridge, UK) or triglyceride quantification assay kit (ab65336, Abcam, Cambridge, UK) according to the manufacturer’s instructions, plasma cholesterol and triglyceride levels were measured using a microplate reader (Sunrise microplate reader, Tecan, Männedorf, Switzerland). 

### 4.3. Histology and Immunohistochemistry

Studies of atherosclerosis by using hyperlipidemic mouse models have focused their investigation on lesions within the aorta or the brachiocephalic trunk (~innominate artery), because they exhibit a highly consistent rate of lesion progression, representing similar atherosclerotic processes occurring in advanced human lesions [33,34,35]. Serial cross sections (7 µm) of BTs were transferred to SuperFrost Plus slides (Thermo Scientific, Waltham, MA USA). Standard hematoxylin-eosin (Carl Roth GmbH + Co. KG, Karlsruhe, Germany) staining was performed on every 10th slide to identify maximum lumen stenosis. Morphometric analyses were performed, like lumen stenosis, neointimal area, media area, and Lillie’s Oil Red O (ORO, Sigma-Aldrich Chemie GmbH, Munich, Germany) staining (to detect lipids). Slides were fixed with isopropanol/1% formalin or 3% phosphate-buffered paraformaldehyde (PFA) solution for immunohistochemistry. Endogenous peroxidase was blocked with 100 U/mL glucose oxidase in PBS for 60 min at 37 °C. Thereafter, a blocking step with 5% porcine serum for 30 min followed and after rinsing with PBS, sections were incubated with primary antibodies for immunohistochemical staining overnight at 4 °C, by using the following antibodies, CD68 (Bio-Rad Laboratories GmbH, Feldkirchen, Germany; MCA1957, dilution 1:200), cyclooxygenase-2 (Abcam, ab15191, 1:800), cleaved caspase-3 (Cell Signaling Technology, Frankfurt, Germany, #9664, dilution 1:100), interleukin-1β (Abcam, ab9722, dilution 1:600), TNF-α (Abcam, ab6672, dilution 1:100). After application of secondary antibody matching primary host species and using ABC method (Vectastain^®^, Vector Laboratories, Inc., Newark, NJ, USA), DAB (Sigma, St. Louis, MO, USA, #D-5906) reaction, added with 0.3% hydrogen peroxide (Roth), was carried out. Afterward, nuclei were stained with Mayer’s hemalum solution, and slides were dehydrated and mounted with Eukitt^®^ (Sigma, #03989).

### 4.4. Histomorphometric Analyses

Serial cross-sections of BTs representing maximum lumen stenosis and maximum plaque formation were photographed at 100-fold magnification. The luminal area, internal elastic lamina (IEL) area, and external elastic lamina (EEL) area were measured with ImageJ/Fiji software version 1.54f (National Institute of Health, Bethesda, MD, USA). The media area is the EEL area minus the IEL area. The neointimal area is the IEL area minus the Lumen area. The area of atherosclerotic lesions, immunoreactivity and lumen stenosis were measured as described earlier [5,6].

### 4.5. Statistical Analyses

Statistical analyses were performed with Sigma Plot 12 (Systat Software Inc., San José, CA, USA). “N” stands for the number of animals per investigated group. Double or triple samples were evaluated per animal. Statistical significance was determined by one-way analysis of variance (ANOVA) with corresponding post-hoc tests. In case the data failed the normality and/or equal variance test, the Kruskall-Wallis and Tukey’s test or Dunn’s method were applied. Results are presented as means ± or standard error of the mean (SEM). The results were considered statistically significant when *p* ≤ 0.05.

## Figures and Tables

**Table 1:** Effect of Maxadilan application on body weight, body weight/tibia length ratio, plasma cholesterol, and triglyceride levels.

	SC		CED	
	Sham	Maxadilan	Sham	Maxadilan
body weight (g)	28.78 ± 0.52	29.41 ± 0.46	29.39 ± 0.59	30.59 ± 0.87
body weight/tibia length (g/mm)	1.52 ± 0.03	1.69 ± 0.02 *	1.38 ± 0.04	1.61 ± 0.06 ^##^
triglycerides (mg/dL)	84.35 ± 16.08	27.94 ± 2.59 *	76.38 ± 19.11	47.49 ± 7.52 ^#^
n	7	8	8	7
total cholesterol (mg/dL)	221.39 ± 23.48	332.93 ± 31.12 *	677.15 ± 105.78	817.09 ± 150.00
free cholesterol (mg/dL)	61.49 ± 7.95	165.41 ± 24.80 ***	302.45 ± 64.37	424.11 ± 77.29
cholesterol esters (mg/dL)	159.90 ± 2.81	167.62 ± 9.37	374.70 ± 24.33	392.98 ± 29.21
n	8	7	7	7

Body weight (g), body weight/tibia length ratio (g/mm). Plasma triglyceride levels, total cholesterol, free cholesterol and cholesterol ester levels in mg/dL were determined by standard spectrophotometric methods. Treated groups received PAC1 agonist maxadilan (20 nmol/kg) and sham physiological saline solution. SC, standard chow; CED, cholesterol-enriched diet; * *p* ≤ 0.05, *** *p* ≤ 0.001 vs. SC Sham mice; ^#^ *p* ≤ 0.05 vs. ^##^ *p* ≤ 0.01 vs. SC maxadilan-treated mice; *p* ≤ 0.001, CED Maxadilan mice vs. SC Maxadilan mice.

## Data Availability

The data sets used and/or analyzed during the current study are available from the corresponding author upon reasonable request.
